# Social Determinants of Stigma and Discrimination in Vietnamese Patients with Chronic Hepatitis B

**DOI:** 10.3390/ijerph16030398

**Published:** 2019-01-31

**Authors:** Thieu Van Le, Thuc Thi Minh Vu, Hue Thi Mai, Long Hoang Nguyen, Nu Thi Truong, Chi Linh Hoang, Son Hoang Nguyen, Cuong Tat Nguyen, Binh Cong Nguyen, Tung Hoang Tran, Bach Xuan Tran, Carl A. Latkin, Cyrus S.H. Ho, Roger C.M. Ho

**Affiliations:** 1 Viet-Tiep Friendship Hospital, Hai Phong 180000, Vietnam; thieulv@gmail.com (T.V.L.); nguyencongbinhvt@gmail.com (B.C.N.); 2 Hanoi Medical University, Hanoi 100000, Vietnam; vuminhthuc2010@yahoo.com.vn (T.T.M.V.); huemt93@gmail.com (H.T.M.); 3 Center of Excellence in Behavioral Medicine, Nguyen Tat Thanh University, Ho Chi Minh City 70000, Vietnam; longnh.ph@gmail.com (L.H.N.); truongnu.ighi@gmail.com (N.T.T.); chi.coentt@gmail.com (C.L.H.); pcmrhcm@nus.edu.sg (R.C.M.H.); 4 Center of Excellence in Evidence-based Medicine, Nguyen Tat Thanh University, Ho Chi Minh City 70000, Vietnam; ng.hoangson29398@gmail.com; 5 Institute for Global Health Innovations, Duy Tan University, Da Nang 55000, Vietnam; cuong.ighi@gmail.com; 6 Department of Lower Limb Surgery, Vietnam-Germany Hospital, Hanoi 100000, Vietnam; tranhoangtung.vd@gmail.com; 7 Institute for Preventive Medicine and Public Health, Hanoi Medical University, Hanoi 100000, Vietnam; 8 Johns Hopkins Bloomberg School of Public Health, Baltimore, MD 21205, USA; carl.latkin@jhu.edu; 9 Department of Psychological Medicine, National University Hospital, Singapore 119074, Singapore; cyrushosh@gmail.com; 10 Department of Psychological Medicine, Yong Loo Lin School of Medicine, National University of Singapore, Singapore 119077, Singapore

**Keywords:** chronic hepatitis B (CHB), hepatitis B virus (HBV), stigma, discrimination

## Abstract

Vietnam is among the countries with the highest prevalence of chronic hepatitis B (CHB) and individuals who suffer from CHB oftentimes perceive high levels of stigma and discrimination. Our study aimed to provide evidence on the prevalence of stigma against hepatitis B virus (HBV), HBV infection, and social determinants of stigma and discrimination in patients. A cross-sectional study was conducted at Viet-Tiep Hospital, Hai Phong, Vietnam. Stigma and discrimination against CHB in the last month were measured via four dimensions: (1) Blame/Judgment; (2) Shame; (3) Discrimination in different settings; (4) Disclosure of CHB status. Multivariate Logistic and Tobit regressions were used to identify factors associated with CHB-related stigma and discrimination. Among 298 enrolled patients, 4.8% experienced blame/judgement, 10.2% perceived shame, 48.5% felt discriminated in healthcare facilities, and 90.6% disclosed their health status with spouses/partners. Factors associated with lower odds of CHB-related stigma/discrimination included living with spouses/partners, old age, being employed, and the existence of comorbidities was linked with higher odds of stigma. Anti-stigma programs should target those who are younger and have comorbidities. This could be done by community-based interventions which focus on inaccurate beliefs about viral hepatitis. Furthermore, families, healthcare providers, and society should play a crucial role in supporting CHB patients.

## 1. Introduction

Chronic Hepatitis B (CHB) is a contagious liver infection caused by the hepatitis B virus (HBV). Globally, in 2015, approximately 257 million people were living with HBV infection and 1.34 million deaths were caused by viral hepatitis [1]. HBV infection has been highly prevalent in developing countries with an estimate of 5% to 15% of all chronic patients [2]. According to the World Health Organization, Vietnam is among the countries with the highest burden of CHB, thus, prompt pragmatic strategies should be prioritized in this country [3].

While the incidence of CHB has been decreased since the universal infant vaccination program against HBV introduced in late 2003 [4], HBV is still highly stigmatized and discriminated against in Vietnam. A study in Ho Chi Minh city, the biggest city in Vietnam, found that 43% of participants would avoid close contact with people who have HBV [5]. The authors claimed that this issue might be due to a low level of vaccination in this population [5]. Another reason could be the misconception regarding hepatitis B transmission routes [5,6]. Although HBV only spreads via blood, sexual intercourse, and mother-to-child pathways, many people are still subconsciously afraid of transmitting the virus through casual contact such as sharing eating utensils, hugging, and kissing [7]. Previous studies in the USA and Canada showed knowledge deficits of how HBV is transmitted in different populations [8,9]. This misconception possibly leads to people with HBV being highly stigmatized [6], oftentimes, being labeled as “lepers” [10] or “AIDS” [11]. Previous literature has endorsed the negative impacts HBV-related stigma on individuals’ behaviors and psychological health [12]. Specifically, people perceiving HBV-related stigma were less likely to screen for HBV [6,13]. Meanwhile, CHB patients fear for the spread of HBV to their family and friends [14]. In addition, stigma may damage CHB patients’ treatment outcomes [12] and quality of life [15].

Currently, available treatments do not cure CHB and the main goal is to prolong survival by slowing down the disease progression and improving quality of life [16]. Therefore, CHB treatment should be parallel with psychological well-being as well as social supports. In Vietnam, less attention has been given to HBV-related stigma [17]. Thus, this study aimed to provide evidence on the stigma against HBV infection and identify the socially vulnerable groups to stigma and discrimination among Vietnamese patients with CHB. The results of this current work should enrich the existing literature and enable relevant stakeholders to implement evidence-based strategies for reducing the negative impacts of HBV in Vietnam.

## 2. Materials and Methods

### 2.1. Study Design and Participants

We conducted a cross-sectional study in October 2018 at the Chronic Hepatitis Clinic in the Viet-Tiep Hospital, Hai Phong, Vietnam. There were approximately 300–330 patients visiting this clinic per month. A convenient sampling method was used to recruit all eligible patients during the study period, who were diagnosed CHB, aged 18 years old or above, and cognitively normal. All eligible patients were asked to give their written informed consent to confirm their participation. From the 309 patients who were invited to participate in the study period, 298 patients accepted to be enrolled into the study (96.4%). The study proposal was reviewed and approved by the Institutional Review Board of Hai Phong University of Medicine and Pharmacy (Code: 128/HDDDBVHNVT).

### 2.2. Data Measurement

Data were collected by face-to-face interviews using a structured questionnaire. First, the research team reviewed the literature to identify which items should be used in this questionnaire. Then, a panel of infectious diseases and public health experts evaluated the appropriate items for this questionnaire. After that, a pilot study with ten patients was performed which tested the compatibility of logical order of questions, language, and social-cultural characteristics of patients. This helped to ensure the reliability and validity of the questionnaire. After receiving feedback from the patients, we revised the questionnaire regarding texts and language. 

The interviewers were medical students at Hai Phong University of Medicine and Pharmacy, who received two careful training sessions conducted by the research team. They also participated in the pilot study as interviewers to understand the way to ask patients questions properly and consistently. Each interview took 15–20 minutes per participant. 

#### 2.2.1. Socioeconomic and Health Status Information

We collected the information regarding age, gender, education, occupation, marital status, and monthly household income (million Vietnam Dong). We also acquired data on the number of comorbidities among the patients. The term “comorbidity” refers to the presence of one or more additional diseases along with CHB. In this study, the data on comorbidities were extracted from patients’ medical records—this data collection method was permitted by Institutional Review Board. Hypertension was the most common comorbidity (28/298 = 9.4%), followed by type 2 diabetes (21/298 = 7.0%).

#### 2.2.2. CHB-Related Stigma and Discrimination

To measure stigma and discrimination, we referred to the Substance Abuse Self-Stigma Scale by Luoma et al. [18], and adapted the conceptual framework by Parker and Aggleton which is used to measure stigma against the MMT (methadone maintenance treatment) and HIV/AIDS population [19]. The previous literature suggested that HIV and HBV infections share various characteristics including transmission routes, social stigma, and impacts on marginalized communities [20]. The final tool included four dimensions: (1) Blame/Judgment; (2) Shame; (3) Discrimination in various settings (workplace, healthcare services, family, and community); (4) Disclosure of CHB status. Respondents were asked whether they had experienced any of the above types of stigma/discrimination in the last month and there were three options in each question: Yes/No/Not. The internal consistency reliability was acceptable with Cronbach’s alpha = 0.712.

1. In general, have you recently been blamed or criticized because of your CHB status? (Item 1)
2. Do you currently feel shame because of your CHB status? (Item 2)
3. Have you felt discriminated against or treated badly by others? In which circumstances (work place/ all health facilities/ family/ community/ others)? (Multiple choice questions) (Item 3)
4. Have you ever disclosed your CHB status to others? With whom did you share (No/Spouses-partners/Parents-Siblings/Relatives/Friends/Health workers/Peers/Others)? (Multiple choice questions) (Item 4)

### 2.3. Statistical Analysis

We used STATA version 15.0 (StataCorp LLC, College Station, TX, USA) to analyze data. A chi-squared test was conducted to compare the difference in the proportion of stigma/discrimination between males and females. Multivariate Logistic and Tobit regressions were used to identify predictors of blame/judgement (Item 1), shame (Item 2), discrimination (Item 3), and the number of disclosed groups (Item 4). For variable “number of disclosed groups”, each response in Item 4 was identified as one disclosed group, resulting in a minimum of zero and a maximum of seven groups that patients could disclose their health status to. Stepwise backward selection strategies with *p* values < 0.2 were utilized to select potential predictors for the reduced models. *p*-value < 0.05 was statistically significant. 

## 3. Results

In a total of 298 respondents, the majority were males (54.5%), nearly 90.0% of respondents lived with spouses/partners, and freelancer was the most commonly reported occupation (36.4%). We found that respondents suffered from different types of stigma: Blame/judgement (4.8%); shame (10.2%). Of note, nearly half of respondents felt discriminated against CHB in healthcare facilities (48.5%) and 90.6% disclosed their health status with spouses/partners. Regarding the number of groups that patients disclosed their health status, the majority shared with more than one group (96.3%) (Table 1).

The predictors of stigma/discrimination are demonstrated in Table 2. Being female and living with spouses/partners were associated with lower risks of perceiving blame/judgement, and those who experienced two comorbidities were more likely to feel blame/judgement than those without comorbidities. In addition, the older group (>60 years old) was less likely to perceive shame in comparison with the younger group (≤30 years old). Notably, employment was significantly associated with higher odds of discrimination than unemployed counterparts.

## 4. Discussion

This current work provided information about CHB-related stigma/discrimination among CHB patients in Vietnam. Despite a low level of perceived blame/judgement and shame, they felt high levels of discrimination in healthcare facilities. Most of CHB patients preferred to disclose their disease status with spouses/partners. Factors associated with lower risks of CHB-related stigma/ discrimination included: Living with spouses/partners; old age; being employed; when comorbidities existence was linked with a higher chance of stigma.

With regards to CHB-related stigmatization, the low prevalence of perceived blame/judgement and shame among CHB patients would, perhaps, indicate the positive perceptions of the general population regarding HBV infection. A previous study in Japan suggested that improving knowledge about HBV may lead to better attitudes towards HBV [21]. Also, the previous literature suggested that the lack of knowledge and awareness about HBV may lead to self-discrimination [12]. However, a prior study in Vietnam found that people with higher knowledge were more likely to have higher degrees of stigma, though this association was not strong [5]. Thus, contextualized community-based education interventions with proper cultural views, focusing on inaccurate beliefs about viral hepatitis, may be an effective approach [5]. Notably, we found the high prevalence of perceived discrimination in healthcare facilities. This discrimination has been highlighted as a great obstacle for accessing services as well as facilitating delays in care among patients with HBV [22]. Therefore, reducing health professionals’ discrimination against those patients, as well as improving their empathy should be considered in the future interventions [22,23]. 

Notably, CHB patients oftentimes disclosed their disease status to spouses/partners. Moreover, we found those living with spouses/partners were at lower risks of CHB-related stigma. Considering the fact that sexual contact is a principal mode of HBV transmission, those who suffer from HBV infection may be afraid of spreading HBV to their spouses/partners, which perhaps could explain their disclosure behaviors [24]. Our study repeatedly endorsed the essential role of spouses/partners and the family as a whole in CHB management. The previous literature consistently acknowledged families as a crucial source of support for those with viral infections, and patients often depend on their primary relationships for supports in the inadequate medical care infrastructure, especially in family-oriented societies like Vietnam [20]. In addition, CHB requires a long-term treatment which may impose a substantial financial burden on patients and their families [25,26]. Furthermore, family supports also play an essential role in promoting psychological and mental well-being among CHB patients. To sum up, our study highlights the role of family supports, particularly spouses/partners, in reducing the self-discrimination among patients with HBV. 

Noticeably, older patients were less likely to perceive CHB-stigma in comparison with their younger counterparts. Undoubtedly, young people are more engaged in social networks via co-workers and friend relationships, thus, HBV infection may result in interrupted social relationships, ineffective working individuals, and increased socioeconomic burdens [27]. As a result, these pressures may reduce self-esteem, leading to the likelihood of perceiving stigma. Furthermore, young people also play an immense part in the viral transmission because of the higher risk of sexual behaviors, drug injection, and sharing needles [28,29,30]. Thus, the existence of CHB may introduce the fear of spreading virus both vertically to the family members and horizontally to the community, which may gradually isolate infected people from the society. Therefore, the future responses aimed at reducing stigma against CHB should focus on younger age groups. 

While previous literature has indicated the negative impacts of discrimination on employment, such as interrupted employability and poor working efficiency [31], our study cited employment as a significant association with CHB-related discrimination. The relationships between discrimination and employment is a dynamically complex process, where discrimination may interrupt people from work and being employed may lead to more discrimination. Discrimination at work has existed for a long time. One potential hypothesis is that those who have jobs highly engage in various social networks and co-worker relationships. Thus, the existence of HBV may violate their inherent dignity and reduce self-esteem, leading to increased discrimination. Since having a stable occupation is almost mandatory to ensure financial resources, a crucial factor for CHB treatment adherence, anti-discrimination policy should be implemented in the workplace. 

Our study also reported the positive association between comorbidities and CHB-related stigma. Apart from liver complications such as cirrhosis, hepatic decompensation, and hepatocellular carcinoma, CHB patients are also at risk of non-liver related medical illnesses [32]. It is important to note that CHB patients experience low quality of life [33], and comorbidities further reduce the quality of life in comparison with the original disorder [34]. Also, since those who suffer from multiple illnesses require more complex medical treatments, they might be at risk of financial catastrophes [35,36]. Moreover, these people highly depend on their family, community, and society, which may result in low self-esteem, self-blame, and self-stigma. Thus, our study found that anti-stigma efforts should target CHB patients with comorbidities. 

To sum up, several important implications were drawn from this study. First, community-based education interventions which focus on inaccurate beliefs about HBV should be extensively implemented in both patients and health workers. By having sufficient knowledge and a positive attitude towards HBV infection, people may reduce stigma and discrimination against CHB. Second, our study highlighted the role of spouses/partners and healthcare provider/social supports as a whole to ensure the elimination of stigma and discrimination. Finally, those who are younger and have multiple illnesses should be the target group for anti-stigma campaigns.

We acknowledged some limitations in this study. First, this sample only engaged patients receiving the CHB treatment in the clinic. Thus, the demographic characteristics in our sample may not reflect these features in the general population. However, the proportion of males observed in this study was comparable with the general Vietnamese population. Accordingly, in 2018, nearly 50% of Vietnamese people were males [37]. Regarding occupation types, while the most common job in the general population is farmers, freelancer was considered as the most popular occupation in this study [38]. Moreover, findings might not be generalizable to those who did not access medical treatments because it could be hypothesized that those perceived higher levels of stigma would not be willing to access to healthcare. Ideally, community-based studies should be designed to capture the comprehensive figure of stigma against CHB. Second, the small sample size could affect the confidence intervals of statistic calculations. Third, recall bias might have occurred as data were self-reported. Finally, the nature of cross-sectional study design could not determine the temporal relationship between independent variables and the outcome of interests. 

## 5. Conclusions

In summary, our study indicated high levels of CHB-related stigma in healthcare facilities. While living with a spouse/partner, old age, and being employed were associated with lower risks of CHB-related stigma, the existence of comorbidities was associated with higher risks of CHB-related stigma. In order to reduce stigma in this group, community-based education interventions which focus on inaccurate beliefs about viral hepatitis should be extensively implemented. Furthermore, we highlighted the crucial role of family, healthcare providers, and society on supporting CHB patients. Finally, the target group for anti-stigma programs should be CHB individuals who are younger and those with multiple illnesses.

## Figures and Tables

**Table 1:** Chronic hepatitis B (CHB)-related stigma and discrimination.

Characteristics	Male		Female		Total		*p*-Value
	n	%	n	%	n	%	
Blame/Judgement	13	8.1	1	0.8	14	4.8	<0.01
Shame	19	11.9	11	8.3	30	10.2	0.31
Discrimination							
Workplace	17	10.5	10	7.4	27	9.1	0.36
Healthcare facilities	82	50.6	62	45.9	144	48.5	0.42
Family	5	3.1	2	1.5	7	2.4	0.36
Community	6	3.7	5	3.7	11	3.7	1.00
Disclosure							
Spouses/partners	148	91.4	121	89.6	269	90.6	0.61
Parents/siblings	40	24.7	32	23.7	72	24.2	0.84
Relatives	137	84.6	116	85.9	253	85.2	0.74
Friends	65	40.1	56	41.5	121	40.7	0.81
Health workers	88	54.3	87	64.4	175	58.9	0.08
Peer educators	4	2.5	6	4.4	10	3.4	0.35
Others	1	0.6	0	0	1	0.3	0.36
Number of disclosed groups *							
1	6	3.7	5	3.7	11	3.7	0.53
2	53	32.7	39	28.9	92	31.0	
3	51	31.5	46	34.1	97	32.7	
4	43	26.5	33	24.4	76	25.6	
5	8	5.0	7	5.2	15	5.1	
6	1	0.6	5	3.7	6	2.0	

* Each response in Item 4 was identified as one disclosed group, resulting in a minimum of zero and a maximum of seven groups that patients could disclose their health status.

**Table 2:** Associated factors with stigmatization and discrimination.

Characteristics	Blame/Judgement		Shame		Discrimination		Number of Disclosed Groups	
	OR	95% CI	OR	95% CI	OR	95% CI	Coef.	95% CI
Gender								
Male (n = 162)	ref		ref		ref		ref	
Female (n = 135)	0.03 **	0.00; 0.57	0.66	0.27; 1.65	0.82	0.47; 1.45	0.02	−0.13; 0.17
Age groups								
≤30 years old (n = 51)	ref		ref		ref		ref	
31–45 years old (n = 69)	4.81	0.18; 125.17	0.33	0.08; 1.32	1.87	0.73; 4.82	−0.07	−0.29; 0.15
46–60 years old (n = 91)	4.52	0.31; 65.91	0.51	0.15; 1.72	0.66	0.28; 1.55	−0.34 ***	−0.57; −0.12
>60 years old (n = 87)	0.16	0.01; 3.03	0.09 ***	0.02; 0.50	0.52	0.20; 1.36	−0.26 **	−0.52; −0.01
Education								
<High school (n = 56)	ref		ref		ref		ref	
High school (n = 125)	1.44	0.20; 10.52	0.96	0.32; 2.90	0.89	0.43; 1.86	−0.01	−0.22; 0.19
>High school (n = 115)	0.11	0.00; 2.64	0.33	0.07; 1.46	0.54	0.22; 1.34	−0.05	−0.29; 0.19
Occupation								
Unemployed (n = 12)	ref		ref		ref		ref	
Freelancer (n = 106)	0.01 ***	0.00; 0.27	0.07 **	0.01; 0.55	11.20 **	1.08; 115.95	0.44	−0.13; 1.01
White-collar workers (n = 59)	0.08	0.00; 2.11	0.10 *	0.01; 1.08	17.91 **	1.58; 203.53	0.43	−0.15; 1.02
Farmer/Blue-collar workers (n = 100)	0.02 ***	0.00; 0.36	0.07 ***	0.01; 0.52	17.37 **	1.67; 180.57	0.42	−0.14; 0.99
Other (n = 14)	0.50	0.02; 14.89			18.53 **	1.39; 247.22	0.49	−0.15; 1.14
Individual monthly income (logarithm)	0.94	0.13; 6.85	0.71	0.18; 2.77	1.34	0.60; 3.01	0.09	−0.12; 0.31
Marital status								
Single/Divorce/Widow (n = 32)	ref		ref		ref		ref	
Having spouse/partner (n = 265)	0.04 ***	0.00; 0.44	0.47	0.15; 1.54	1.53	0.60; 3.89	0.18	−0.07; 0.44
Number of comorbidities								
0 (n = 155)	ref		ref		ref		ref	
1 (n = 89)	3.88	0.69; 21.77	1.20	0.45; 3.21	0.51 **	0.28; 0.95	0.06	−0.10; 0.23
2 (n = 54)	9.44 **	1.11; 80.04	1.43	0.35; 5.81	0.41 **	0.18; 0.91	0.02	−0.20; 0.24

* *p* < 0.1; ** *p* < 0.05; *** *p* < 0.01.
